# Hysteretic Conductance in Ion Channel Gating

**DOI:** 10.3390/e28060650

**Published:** 2026-06-09

**Authors:** Bartek Lisowski, Martin Bier, Bartłomiej Dybiec, Ewa Gudowska-Nowak

**Affiliations:** 1 Chair of Pharmaceutical Technology and Biopharmaceutics, Faculty of Pharmacy, Jagiellonian University Medical College, ul. Medyczna 9, 30-688 Kraków, Poland; bartek.lisowski@uj.edu.pl; 2 Department of Physics, East Carolina University, Greenville, NC 27858, USA; bierm@ecu.edu; 3 Institute of Theoretical Physics and Mark Kac Complex Systems Research Center, Jagiellonian University, ul. S. Łojasiewicza 11, 30-348 Kraków, Poland; bartlomiej.dybiec@uj.edu.pl

**Keywords:** channel gating, nonequilibrium and irreversible thermodynamics, fluctuation phenomena

## Abstract

Hysteresis seems to play a critical role in the generation and modulation of electrical signal events in neurons, muscles, and other excitable tissues. In voltage-gated ion channels, hysteretic conductance manifests under cycling changes in transmembrane voltage when conductance is delayed in response to voltage changes. Such dynamic behavior emerges naturally when the frequency of the oscillatory voltage becomes comparable to the characteristic relaxation time associated with transitions between channel conductance states and is reminiscent of hysteresis observed in transistors, memristors or solar cells. To investigate this delayed response, various discrete-state Markov models have been proposed. In these frameworks, an ion channel is represented as a finite set of states—typically corresponding to closed and open conformations—with transitions governed by voltage-dependent rates. As an alternative, the progress of activation and transition between opening and closing states of a channel is described in terms of a diffusive, collective “reaction coordinate” which fulfills a Langevin equation and the Smoluchowski–Fokker–Planck equation associated with it. Here we review this approach in modeling dynamic memory of ion channels.

## 1. Introduction

Ion channels are transmembrane, pore-forming proteins which regulate ionic currents through the cell membrane and undergo conformation deformations under environmental (temperature, electric field, pressure) changes. At the level of a single channel the gating process, which changes permeability of ions in response to voltage change across the cell membrane, also incorporates local conformational variations of the constituting proteins [1]. Hysteresis, termed otherwise “mode shift”, in ion channels is a phenomenon in which a conductance loop arises in delayed response to voltage change, thus exhibiting a memory effect. Such hysteretic current–voltage characteristics have been detected in various biological channels [2,3,4,5,6,7,8,9] and the physiological significance of the phenomenon has been debated over the years [10,11,12,13,14,15].

A cornerstone of modern electrophysiology is the patch clamp technique, which enables direct measurement of ionic currents through individual ion channels in biological membranes [16]. Developed in the late 20th century, it uses a glass micropipette to form a high-resistance seal (“gigaseal”) with a small patch of membrane. This configuration allows for precise control of the membrane potential and the recording of extremely small currents, often in the picoampere range. Variants such as cell-attached, whole-cell, inside-out, and outside-out configurations provide flexibility for studying ion channel behavior under different physiological conditions.

Single-channel kinetics refers to the analysis of the stochastic opening and closing (gating) behavior of individual ion channels as recorded by a patch clamp [17,18]. The channels do not conduct ions continuously but instead switch between some discrete states (e.g., open, closed, inactive). By analyzing dwell times (how long a channel remains in a given state) and transition probabilities, channel behavior can be modeled using kinetic schemes, often based on Markov processes. This approach reveals key properties such as conductance, open probability, and the effects of ligands, voltage, or mutations on channel kinetics and function.

Together, patch clamp recording and single-channel kinetic analysis have provided deep insights into the fundamental mechanisms of excitability, synaptic transmission, and signal transduction. They remain essential tools in neuroscience, cardiology, and pharmacology, particularly for understanding disease-related channel dysfunctions and for drug development targeting ion channels.

In this review, we examine the theoretical frameworks used to model the dynamic memory of voltage-gated ion channels. We begin by discussing discrete-state Markov models, which utilize Boltzmann statistics to describe transitions between channel conformations. Following this, we present a deterministic toy model that bridges conformational kinetics with memristive theory, characterizing the ion channel as a “sluggish resistor” where the current state is a cumulative reflection of its preceding electrical history. To provide a more realistic description of activation kinetics, we further explore Brownian models that represent gating as a stochastic diffusion process—governed by Langevin and Smoluchowski–Fokker–Planck equations—within a voltage-dependent energy landscape. Finally, we consider the influence of pore geometry on entropic barriers and investigate how cooperative gating in localized clusters leads to collective dynamics that cannot be captured by independent gating models.

## 2. Discrete-State Models of Gating Kinetics

Understanding of individual channel gating kinetics comes mostly from experiments based on the on-cell patch clamp technique which allows the recording of currents due to one or a few stochastically gating single channels in response to a command voltage [19]. A common two-state model Equation (6) is a simplification of more detailed models in which there can exist many inactivated states and multiple subunits, each of which can be in an open state, and the channel conducts when all subunits are open. The mechanism of the gating process is then commonly described by discrete Markov models [20] represented by kinetic schemes incorporating multiple open/inactivated states connected by (fixed) rate constants [21,22].

The essence of this approach, formulated in an early work of Hodgkin and Huxley [23], is based on applying the Boltzmann statistics to describe the probability of a channel occupying a given conductance state, including the voltage dependence of this probability.

For the purpose of modeling two-state channel kinetics, one associates an internal energy $E_{O}$, $E_{C}$ with each of the two states, and assumes that transitions between the states are thermally activated barrier crossings. The probability that a channel is open ($P_{O}$) or closed ($P_{C}$) is

(1) $$P_{O,(C)}=\frac{e^{-E_{O,(C)}}}{e^{-E_{O}}+e^{-E_{C}}}=\frac{1}{1+e^{\pm \Delta E}},$$

where all energies are given in units of $k_{B}T$. In the case of voltage-gated channels, the transition between the open and the closed states involves movement of charges in the transmembrane field, reorientation of local polarization and conformational variation of the channel protein that results in the change in energy ΔE of the system. In the simplest approximation, this energy difference is assumed to depend linearly on the membrane voltage, reflecting the work performed by the effective gating charge moving across the transmembrane electric field, so that $\Delta E\propto V-\bar{V}$. While the linear approximation is standard and successfully captures the primary charge-movement mechanics in many voltage-gated channels, it is not universally applicable. In physiological scenarios involving significant membrane deformation, complex electrostatic interactions, or coupled gating transitions, non-linear voltage dependencies can emerge, requiring more elaborate parametrizations of ΔE [24,25].

With ΔE as above, Equation (1) may be written as

(2) $$P_{O}(V)=\frac{1}{1+e^{-\alpha (V-\bar{V})}}\equiv f(V),$$

where $\alpha \bar{V}$ replaces $\left(E_{C}-E_{O}\right)/k_{B}T$, $\bar{V}$ is the voltage at which $P_{O}=P_{C}=1/2$ and αV parameterizes all other contributions to ΔE.

In models of a voltage-gated ion channel characterized by two conformational states, spontaneous opening and closure events are described by a process similar to an activated uni-molecular chemical reaction pictured by a kinetic scheme

(3) $$\mathrm{open}\begin{array}{c} k_{C}(t)\\ ⇌\\ k_{O}(t) \end{array}\mathrm{closed}.$$

The ionic current I(t) of the channel molecule is then expressed as

(4) $$I(t)=G\left(P_{O}(t),V(t),t\right)V(t)$$

where function *G* stands for

(5) $$G(t)=[g_{C}\left(V(t)\right)+\left(g_{O}\left(V(t)\right)-g_{C}\left(V(t)\right)\right)P_{O}(t)],$$

with $g_{O,C}$ denoting conductances of ion-conducting (open) and ion-nonconducting (closed/inactivated) states and $P_{O}(t)$ expressing the probability of finding the channel in an open state [6,11,26,27,28]. Note that in general, the conductance may be voltage- and time-dependent. According to the scheme Equation (3) the time evolution of $P_{O}(t)$ can be captured by the Markovian two-state process

(6) $$\frac{dP_{O}(t)}{dt}=-k_{C}(t)P_{O}(t)+k_{O}(t)\left[1-P_{O}(t)\right]$$

in which the kinetic rates $k_{O}(t)=k_{O}^{*}\exp\left[\alpha V(t)\right]$ and $k_{C}(t)=k_{C}^{*}\exp\left[-\beta V(t)\right]$ are voltage-dependent and describe stochastic transitions between open (O) and closed (C) states. The solution to the above rate equation is

(7) $$P_{O}(t)=P_{O}\left(t_{0}\right)e^{-\int_{t_{0}}^{t}k\left(t_{1}\right)dt_{1}}+\int_{t_{0}}^{t}k_{C}\left(t_{1}\right)e^{-\int_{t_{1}}^{t}k\left(t_{2}\right)dt_{2}}dt_{1},$$

with $k(t)=k_{O}(t)+k_{C}(t)$. For a periodic voltage V(t+T)=V(t) with nT<t<(n+1)T one can use a recursion formula derived from Equation (7); see [6,27]:

(8) $$P_{O}\left(\left(n+1\right)T\right)=\kappa P\left(nT\right)+c_{0},$$

where κ and $c_{0}$ refer to

(9) $$\kappa =\exp\left[-\int_{0}^{T}k(t)dt\right]$$

and

(10) $$c_{0}=\int_{0}^{T}k_{C}\left(t_{2}\right)\exp\left[-\int_{t_{2}}^{T}k\left(t_{1}\right)dt_{1}\right]dt_{2}.$$

Equation (8) can be used to express $P_{O}\left(nT\right)$ in terms of $P_{O}\left(0\right)$ which stands for the equilibrium open probability in the absence of voltage (V(t=0)=0); i.e., $P_{O}\left(0\right)=k_{C}^{*}/\left(k_{O}^{*}+k_{C}^{*}\right)$:

(11) $$P_{O}\left(nT\right)=\kappa^{n}P_{O}\left(0\right)+\frac{1-\kappa^{n}}{1-\kappa }c_{0}.$$

For n→∞ the above formula approaches $c_{0}/\left(1-\kappa \right)$ and used with Equation (7) yields the asymptotic probability

(12) $$P_{O,\infty }=\lim_{n\to \infty }P\left(t|n\right)=\frac{c(t)}{1-\kappa }$$

with function c(t) given by

(13) $$c(t)=\int_{t}^{t+T}k_{C}\left(t_{2}\right)\exp\left[-\int_{t_{2}}^{t+T}k\left(t_{1}\right)dt_{1}\right]dt_{2}.$$

Accordingly, the asymptotic open probability Equation (13) is periodic and may exhibit a hysteresis loop in the ($P_{O,\infty }(t),V(t)$) plane. The loop area is expressed in terms of the cyclic integral of the system’s response to periodic modulation $A_{\mathrm{hyst}}=\int_{0}^{2\pi /\omega }dV(t)\langle P_{O}(t)\rangle$ with an average $\langle P_{O}(t)\rangle$ taken over multiple cycles of V(t)=V(T+t) with T=2π/ω,

(14) $$A_{\mathrm{hyst}}=\int_{0}^{T/2}P_{O,\infty }\left(t)\right|\dot{V}(t)|dt-\int_{-T/2}^{0}P_{O,\infty }(t)\left|\dot{V}(t)\right|dt$$

and exhibits a maximum at some intermediate voltage frequency and disappears in the limits of low and high frequencies. Experimental protocols used to reveal voltage-dependent gating mechanisms [1,29] usually impose a cyclic ramp in the control parameter V(t). As explained above, the key consequence of this driving is that adaptation of the channel protein to a stimulus is not instantaneous but requires finite relaxation times and may produce a delayed response at nonzero sweep rates. This delay leads to distinct up- and down-sweep branches of a gating current I(t), yielding a nonzero dynamic hysteresis loop area. When the voltage varies sufficiently slowly, the protein constituent of the channel has enough time to adjust its conformation to the instantaneous value of the voltage. As a consequence, the ion current through the channel is independent of the prehistory and hence no hysteresis is observed. On the other hand, when the period of the voltage change is much shorter than the characteristic protein relaxation time, the protein molecule cannot follow fast variations in the voltage and adapts only to its average value. As a consequence, the current through the channel becomes again independent of the former history, and the hysteresis loop collapses to a single line. Altogether, the loop area first grows monotonically with the frequency of voltage change, reaches a maximum, and disappears as the frequency tends to infinity [28,30,31,32].

Finally, we mention that Equations (4) and (6) are reminiscent of widely investigated “memory devices”, including the Hodgin–Huxley model that describes the temporal dynamics of action potentials in neurons [5,8,33], and are frequently used within the framework of Chua’s theory of memristive systems [34,35,36,37].

To formalize the connection between conformational kinetics and the theory of memristive systems, the following subsection presents a deterministic toy model that illustrates the mechanism behind the emergence of macroscopic current–voltage hysteresis.

### Deterministic Toy Model: The Ion Channel as a Memristive System

**Figure 1:**
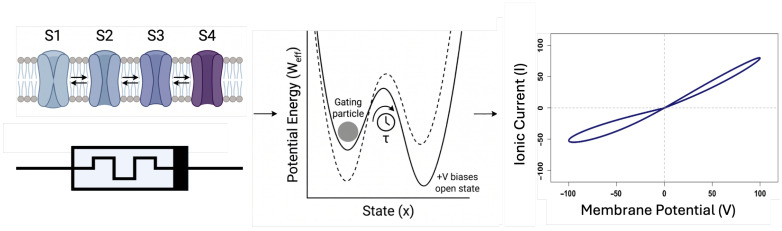
(**Left**) Schematic of the gating process represented as a progression through a chain of conformational states ($S_{1}⇌S_{2}⇌S_{3}⇌S_{4}$), mapped to a collective reaction coordinate S(t). The memristor symbol highlights the functional analogy between biological gating and memory-based electronic components. (**Middle**) The effective potential landscape $W_{\mathrm{eff}}\left(x;V\right)$, where the external voltage biases the energetic stability of open versus closed configurations. The relaxation time constant τ=13.0 a.u. introduces a kinetic lag, representing the protein’s “sluggish” adjustment to the instantaneous thermodynamic target $S_{\infty }(V)$, as described in the main text. (**Right**) Pinched hysteresis loop in the I−V plane, resulting from a toy model simulated with a periodic sinusoidal driving force ($V_{\max}=100$ a.u., ω=0.15). The smooth, symmetric “bow-tie” characteristic arises from the sigmoidal Boltzmann equilibrium (α=0.06) and the linear mapping of the state variable to conductance ($g_{\min}=0.2$, $g_{\max}=1.0$).

Following the theory of memristors and its applications to biological systems [35,38,39,40], an ion channel can be described as a non-linear conductor where the instantaneous current I(t) depends on the current conductance state *G*, which in turn is conditioned by the history of the transmembrane voltage V(t), cf. Equation (4). In the proposed deterministic model, the gating process is represented by a continuous reaction coordinate S(t)∈[0, 1], describing the progress of the channel protein’s conformational changes [14,41,42], as shown in Figure 1. The system dynamics is based on the relationship between current and voltage:

(15) I(t)=G(S(t))V(t),

where the conductance G(S) scales linearly between a minimum value ($g_{\min}$) and a maximum value ($g_{\max}$),

(16) $$G(S)=g_{\min}+\left(g_{\max}-g_{\min}\right)S(t).$$

The thermodynamic equilibrium state, $S_{\infty }(V)$, which the protein approaches under the influence of the applied potential, is described using Boltzmann statistics. The use of a sigmoidal activation function allows for smooth saturation of the channel response at extreme voltage values:

(17) $$S_{\infty }(V)=\frac{1}{1+e^{-\alpha (V-\bar{V})}},$$

with $\bar{V}$ standing for the midpoint determining the voltage at which the equilibrium state reaches half activation, i.e., $S_{\infty }\left(\bar{V}\right)=1/2$. The key element generating the memory effect, signaled by the hysteresis, is the finite relaxation time τ. The time evolution of the state variable is described by a deterministic relaxation equation:

(18) $$\frac{dS(t)}{dt}=\frac{S_{\infty }\left(V(t)\right)-S(t)}{\tau }.$$

Assuming a periodic voltage driving force $V(t)=V_{\max}\sin(\omega t)$, the delay in the conformational response relative to the stimulus, τ, leads to the emergence of a characteristic pinched hysteresis. This mechanism essentially characterizes the ion channel as a “sluggish resistor”—a term referring to a resistive element whose conductance state cannot adapt instantaneously to voltage fluctuations due to internal kinetic barriers. This “sluggishness” is the physical basis for the system’s dynamic memory, as the phase lag between the stimulus and the response ensures that the current state of the channel is a cumulative reflection of its preceding electrical history rather than just the instantaneous potential.

The signature zero-crossing point in the V−I plane, used since the pioneering work of Chua as the fingerprint of a true memristor, is a feature of a passive memristor that, unlike a capacitor or an inductor, does not store energy. The pinch occurs because when the applied voltage forces the net driving potential to zero, the current flowing through the open channel also drops to zero.

A pinched hysteresis loop is not universally typical [43] for all biological channel hysteresis, but it is the defining characteristic of a specific subset of voltage-gated ion channels behaving as ideal biological memristors.

It is interesting to note that the area of the resulting hysteresis loop represents, using the term proposed by Millar in [44], the *content* of the memristive system: a physical quantity with a dimension of power that is formed solely by the contribution of memory effects rather than power-energetic phenomena. As the instantaneous power is zero at the end of each half-period, this area serves as a pure quantitative indicator of the system’s instantaneous memory state [45,46].

While this deterministic framework effectively captures the collective “memristive” signature of the channel, it abstracts away many known biophysical features, such as the stochastic fluctuations inherent to individual protein molecules [47,48]. To provide a more sophisticated and realistic description of activation kinetics, the following section introduces Brownian models, where gating is represented as the stochastic diffusion of a collective reaction coordinate within a voltage-dependent energy landscape.

## 3. Brownian Models of Gating

A classical theoretical approach represents gating using a small number of internal degrees of freedom [11,12,49,50], typically combining a fast gating variable with a slow conformational coordinate that evolves on a longer time scale and thereby generates hysteresis. Such low-dimensional models provide an intuitive energetic picture of memory and they typically encode hysteresis via an assumed effective landscape or explicitly postulated slow coordinates [27,32,51,52,53,54].

More realistic descriptions of the activation kinetics of a voltage-dependent channel incorporate models of the Brownian motion of a fictitious “gating particle” diffusing in an energy landscape reflecting coupling of gating kinetics to conformational changes in the structure of the protein forming the channel [52,55,56,57]. This approach offers a description in which the configurational change of the channel protein is modeled by a multi-component “reaction coordinate” *x*. Since the potential energy of the entire system (the gate, channel protein and membrane environment) is a function of hundreds of relevant coordinates of the system, *x* can be understood as a collective variable similar to its definition in the Marcus theory of charge-transfer reactions [58]. When *x* can be viewed as a reliable measure of the progress of the gating reaction, the open and closed states of a channel can be mapped to stationary states of a one-dimensional potential of the mean force [52,56,57,59]. Computational tests based on atomistic models of channels derived from crystallographic data have confirmed the rationale of this approach [47,57] which allows the construction of a model of gating based on Kramers’ theory of activated processes. The phenomenological description relies on a double-well potential with minima denoting open/closed states respectively, with a single potential (activation) barrier separating these two regions on the *x*-axis.

**Figure 2:**
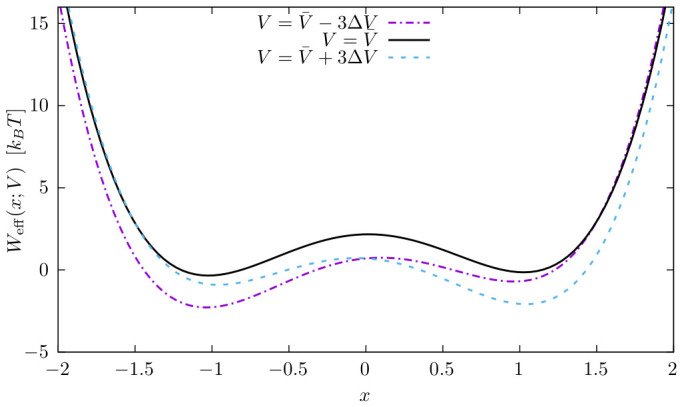
The effective potential for conformational changes, $W_{\mathrm{eff}}\left(x;V\right)$, for $V=\bar{V}$, $V=\bar{V}+3\Delta V$ and $V=\bar{V}-3\Delta V$. Parameters of the potential (in units of $k_{B}T$) $\Delta E_{\mathrm{conf}}=0.6$, $\Delta E_{\mathrm{gate}}=9.72$, $\Delta E_{\mathrm{int}}=2.16$, $x_{\max}=0.018$, ΔV=12 and $\bar{V}=66$. The set of parameters has been chosen to model hysteretic behavior observed in opening probabilities in the human erythrocyte voltage-dependent cation channel [4,52].

In order to capture the configurational change of the protein quantitatively, we introduce a parameterization for the reaction coordinate *x* for this change by assuming that the equilibrium probability of the system being in a conformational state *x* is given by

(19) $$p(x)=\sum_{\epsilon =\pm 1}p\left(\epsilon ,x\right)=e^{-W_{\mathrm{eff}}\left(x;V\right)},$$

where ϵ describes a binary variable ϵ=±1 discriminating between open (ϵ=1) and closed (ϵ=−1) states of the channel. A corresponding effective potential (cf. Figure 2) takes on the form [31,52]

(20) $$W_{\mathrm{eff}}\left(x;V\right)=W(x)-\log\left[\cosh\left(\frac{1}{2}\left(\Delta E_{\mathrm{gate}}+\Delta E_{\mathrm{int}}x\right)\right)\right],$$

where W(x) describes the protein’s internal energy as a function of *x*,

(21) $$W(x)=\Delta E_{\mathrm{conf}}\left(\frac{1}{4}x\left(3-x^{2}\right)+\frac{3}{16x_{\max}}{\left(1-x^{2}\right)}^{2}\right),$$

with $\Delta E_{\mathrm{conf}}$ being a measure of the difference in the internal energy associated with the change in the protein’s configuration favoring open (closed) states of the gate. The parameter $x_{\max}$ has been chosen as the value of the reaction coordinate *x* at which the local maximum, the top of the barrier between x=±1 states, is located; $\Delta E_{\mathrm{gate}}=-\alpha \left(V-\bar{V}\right)$ is the contribution to the internal energy discriminating between the open (closed) states of the gate and $\Delta E_{\mathrm{int}}=-\alpha \Delta V$ describes interaction energy between the gate and surrounding protein. In the above formula ΔV stands for the maximum intensity of the driving external voltage *V* and $\bar{V}$ is an average value of *V* for which the stationary probability $P_{O}$ achieves a value of 1/2.

We now additionally assume that the membrane potential felt by a channel is not exactly constant, but changes, albeit slowly as compared to the rates of the gate opening and closing

(22) $$V(t)=\bar{V}-\Delta V\cos\Omega t.$$

**Figure 3:**
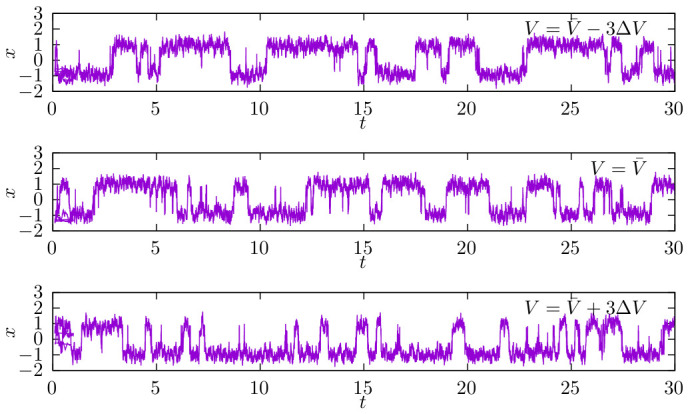
Sample realizations of the stochastic process X(t), for the two-state and the continuous models, derived from numerical simulations of Equation (24). The time step of simulations was set up to $10^{-3}$ with the frequency of the cycling voltage Ω=1. Trajectories were run for the duration time T≈10π. The initial value of *X* was sampled from the uniform distribution on the interval [−1.5,1.5].

For large positive $V(t)-\bar{V}$, stability analysis shows that conformational state x=1 is favored over x=−1 with an energy difference $\alpha \Delta V=|\Delta E_{\mathrm{int}}|$. Similarly, for large negative $V(t)-\bar{V}$

(23) $$W_{\mathrm{eff}}\left(x;V\right)=W(x)+\frac{1}{2}\alpha \left(V-\bar{V}+\Delta Vx\right)+\log2$$

and the barrier at $x_{\max}$ becomes lowered by $\frac{1}{2}\alpha |V-\bar{V}|=\frac{1}{2}\left|\Delta E_{\mathrm{gate}}(V)\right|$ favoring state x=−1 over x=1. Note that for large differences $V(t)-\bar{V}$, addition of time-dependent driving does not change characteristic relaxation frequencies of the system within the wells of the potential $W_{\mathrm{eff}}(x)$. Direct evaluation of $\omega_{x_{\max}}^{2}=W_{\mathrm{eff}}^{\prime \prime }\left(x_{\max}\right)$ and $\omega_{\pm 1}^{2}=W_{\mathrm{eff}}^{\prime \prime }\left(x=\pm 1\right)$ yields $\omega_{x_{\max}}^{2}\approx -3\Delta E_{\mathrm{conf}}/4$, $\omega_{\pm 1}^{2}\approx 3\Delta E_{\mathrm{conf}}/\left(2x_{\max}\right)$ for values of $x_{\max}$ close to 0. We further assume that fluctuations in the collective variable *x* quickly equilibrate; i.e., their dynamics can be well approximated by an overdamped Langevin equation

(24) $$\frac{dx}{dt}=-\frac{dW_{\mathrm{eff}}\left(x;V\right)}{dx}+\xi (t),$$

with ξ(t) representing effects of thermal noise modeled by uncorrelated Gaussian white noise—i.e., $\langle \xi (t)\xi \left(t^{\prime }\right)\rangle =\sigma^{2}\delta \left(t-t^{\prime }\right)$. For convenience, we set the friction coefficient γ=1 which rescales the time *t* in the above equation. Figure 3 presents samples of stochastic trajectories X(t) simulated from the above Langevin equation at constant intensity $\sigma^{2}=8$ of the additive noise (mimicking conditions of a constant temperature of the thermal medium). They model records of current in a biological channel switching between conducting and non-conducting conformations.

The response of the ensemble average coordinate 〈x(t)〉 to a mixture of fluctuations and external periodic perturbation can be otherwise described by the Smoluchowski–Fokker–Planck equation associated with Equation (24):

(25) $$\frac{\partial }{\partial t}p\left(x,t\right)=\frac{\partial }{\partial x}\left[p\left(x,t\right)\frac{\partial W_{\mathrm{eff}}}{\partial x}\right]+\frac{\sigma^{2}}{2}\frac{\partial^{2}}{\partial x^{2}}p\left(x,t\right).$$

By defining the occupation probability of the open state as

(26) $$P_{O}(t)\equiv \mathrm{Prob}\left\{x(t)<x_{\max}\right\}=\int_{-\infty }^{x_{\max}}p\left(x,t\right)dx=1-P_{C}(t)$$

one can analyze the time-dependent open probability from simulated trajectories x(t).

As expected from the former analysis, at large positive values of the gating voltage *V*, the channel is predominantly open with current fluctuating due to the local conformational variations around the open state (x=1). At $V=\bar{V}$ none of the states is favored, whereas for large negative *V* a preference for staying in a closed state is visible. Increasing the noise intensity at a constant value of the voltage cycling frequency causes the trajectory to fluctuate wildly between both wells of the effective potential, shortening the time the process spends in the vicinity of the metastable points x=±1 and eventually destroying the pattern of the signal switching between “open” and “closed” states.

**Figure 4:**
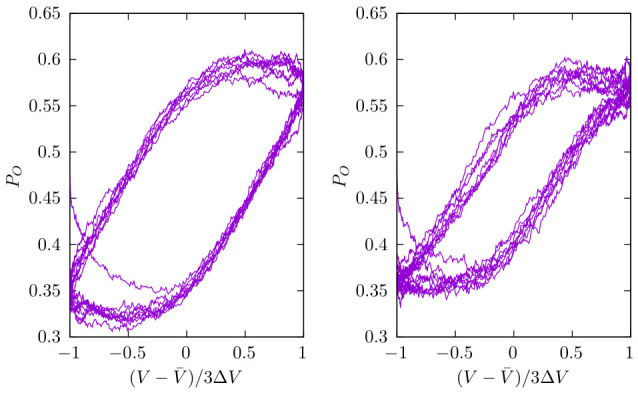
The open state probability $P_{O}$ as a function of the membrane potential $(V(t)-\bar{V})/3\Delta V$. Probability has been estimated from the frequency histograms for $3\times 10^{3}$ trajectories mimicking passages between different conductance levels. Intensity of the thermal noise $\sigma^{2}=6$ (**left**) and $\sigma^{2}=8$ (**right**); time step of the simulation $dt=10^{-3}$.

From the residence time frequency histograms for the open and closed states, we have replotted the probability of the channel being open as a function of the driving voltage. Figure 4 presents estimated opening probabilities for various intensities of thermal noise. Note that unlike the current–voltage hysteresis discussed in Section Deterministic Toy Model: The Ion Channel as a Memristive System and depicted in Figure 1, the loop in Figure 4 is presented as a conductance hysteresis curve in the (G,V) (or, equivalently, in the $\left(P_{O},V\right)$) plane.

By increasing the activating voltage from $V=\bar{V}$ to $V=\bar{V}+3\Delta V$ and reversing the direction of voltage changes to $V=\bar{V}-3\Delta V$, an apparent hysteretic shape of the $P_{O}(V)$ curves is observed (cf. Figure 4). The hysteresis changes its shape and area with increasing intensity of the thermal noise and disappears for large values of $\sigma^{2}$. Through cycling V, the height of the potential barrier dividing wells of open and closed states is itself a time-dependent variable. From other contexts [26,60,61,62,63] it is known that such an interplay of periodic forcing and additional noise may lead not only to dynamic hysteresis but also to other nontrivial effects like stochastic resonance or resonant activation.

In one cycle of voltage modulation the internal energy of the system does not change, so that by the first law of thermodynamics, the work associated with a sweeping voltage is transferred into dissipated heat which is represented by the loop area $A_{\mathrm{hyst}}$. The latter also provides a quantitative measure of memory in the gating dynamics. Parametric plots of the cumulative open probability as a function of voltage display a loop whose shape and area depend on the overall relaxation rate $k(t)=k_{O}(t)+k_{C}(t)$. The hysteresis disappears only in the case of driving that has a time scale significantly different from the inverse of rates $k_{O,C}$.

Interestingly, the paradigm of dynamic memory can also be recovered in single-well models of voltage switching in conductance (cf. Equation (24)). In a harmonic well, a signal-enhancing effect—observed as a form of stochastic resonance—and a hysteretic loop in response can be registered under the action of two correlated noises: a multiplicative ξ(t) with intensity *p* and an additive $\xi_{a}(t)$ with intensity *q* [37,64]:

(27) $$\frac{dx}{dt}=-\left(a+p\xi (t)\right)x+V_{0}+q\xi_{a}(t)+V_{1}\cos(\omega t+\phi ),$$

where $\xi_{a}(t)$ is given by a combination of Gaussian white noises

(28) $$\xi_{a}(t)=c\xi (t)+\sqrt{1-c^{2}}\eta (t),\langle \xi (t)\eta \left(t^{\prime }\right)\rangle =0.$$

For $V_{0}p-acq=0$ the above system exhibits a pinched hysteresis loop in the $\left(x,V_{1}\cos\omega t\right)$ plane and stochastic resonance in the signal-to-noise ratio [37,64].

Brownian motion models have been extensively used, especially after elucidation of the crystal structure of voltage-gated channels. They allowed molecular dynamics modeling to come into play to properly identify fields of effective forces $-dW_{\mathrm{eff}}\left(x;V\right)/dx$ entering the overdamped dynamics in Equation (24).

In a series of papers [7,8,16] discussing the use of molecular dynamics to simulate a voltage sensor along its activation pathway, the energy landscape governing the sensor’s position was derived, and the electrostatic potential profile was computed using the Poisson equation, taking into account all charges present in the system.

## 4. Channel Pore Geometry, Entropic Effects and Cooperative Gating

Over the past years several studies have concentrated on the impact of pore geometry on the rules for gating by shaping energy barriers. Wider cavities in membranes are expected to stabilize ions and water, lowering the barriers for conduction and speeding up channel opening, whereas small changes in radius can switch the pore from conductive to non-conductive [65,66,67,68]. To estimate a change in entropy resulting from the change in channel pore geometry during membrane depolarization, the authors in [65,67,69,70] used the concept detailed in [71] that the spatial confinement causes an entropic barrier. In this approach changes in entropy stem from the differences in volume of the configuration space available for passing particles within the channel interior at different voltages. In the model, ion channels were considered as quasi-one-dimensional structures with ions moving along the transmembrane channel axis. Results of simulations of an ion random walk in entropic and electric potentials indicated that the tendency to occupy open states at membrane depolarization is entropy-facilitated which is an important factor contributing to the observed single-channel currents.

Contemporary techniques that directly visualize cellular membranes, like confocal fluorescence microscopy, challenge the concept of independent channel gating. Channels in biological membranes often organize into spatially localized clusters, where they can exhibit cooperative gating behavior [15,72]. In such systems, the activity of an individual channel influences the opening probability of neighboring channels [47,73,74], giving rise to collective dynamics that cannot be explained by independent gating models. Understanding these inter-channel interactions is essential for elucidating the molecular basis of electrochemical signaling and for developing targeted pharmacological interventions.

In the study [73], the authors introduced a simplified stochastic model of multi-channel gating designed to systematically investigate cooperative effects under controlled conditions. The model captures key features of inter-channel coupling while remaining analytically and computationally tractable, enabling the exploration of how interaction strength, cluster size, and intrinsic channel kinetics shape emergent gating behavior. Two information-theoretic metrics, i.e., Shannon entropy and Sample Entropy, were applied to simulated multi-channel datasets, including idealized total current traces and dwell-time sequences of cluster states, to quantify inter-channel cooperativity. Both entropic measures were shown to display a strong dependency on the strength and type of cooperation (non-, positive, or negative cooperation).

In particular, recent studies by Suma et al. [47] have shown that fluctuations in the local lipid composition generate effective attractive interactions and induce correlations between ion channels, even at large separations. These effects underlie the observed tendency of channels to cluster and their cooperative activation. Moreover, because the characteristic relaxation times of these fluctuations exceed the time scales associated with channel activation, the channel dynamics exhibits memory effects, including hysteresis in the activation curve.

Altogether, individual channels and lipid molecules were shown to selectively interact with specific sites on integral membrane proteins, and modulate their structure and function. Identification and characterization of these sites are of importance for understanding of not only the molecular basis of membrane protein function and stability, but also the mechanism of biological channel gating [47,75].

## 5. Conclusions

Molecular dynamics simulations now enable quantitative analysis of ion channel conduction, activation, and drug modulation, bridging the gap between static structural data and dynamic function. These techniques [76] combine long-time-scale simulations with enhanced sampling methods to provide atomistic insights into channel mechanisms and to identify new, targeted pharmacological sites.

Equally intriguing are recent advances in artificial solid-state nanopores and nanochannels, which can be regarded as structural and functional mimics of protein ion channels. While these architectures have been successfully developed for sensing applications [77,78], gate-controlled ionic transitions have also stimulated tremendous contemporary interest in creating artificial systems for neuromorphic computing, an idea pioneered by Carver Mead [79]. Inspired by biological gating, hysteresis, and synaptic plasticity, these systems exploit controlled ion currents to process information directly in liquid media [80]. Although a detailed analysis of this rapidly expanding field falls outside the scope of the present article, it is important to emphasize that understanding the fundamental biophysics of biological ion channels directly informs the design of next-generation neuromorphic ionic architectures (for comprehensive reviews, see [81,82]). More broadly, the integration of biological data with simplified physical and mathematical models has made it possible to explain gating mechanisms, interpret experimental observations, and generate new insights into ion channel function.

## Data Availability

No new data were created or analyzed in this study.
